# Dendritic Cell Migration: An Essential Step in Initiating Adaptive Immunity Across Tissues

**DOI:** 10.1111/imr.70080

**Published:** 2025-11-30

**Authors:** Eli C. Olson, Adam Williams, Stephanie C. Eisenbarth

**Affiliations:** ^1^ The Department Medicine, Division of Allergy and Immunology Northwestern University Feinberg School of Medicine Chicago Illinois USA; ^2^ Center for Human Immunobiology Northwestern University Feinberg School of Medicine Chicago Illinois USA

**Keywords:** cytoskeleton, dendritic cells, immune surveillance, intestine, lung, lymph node, migration, skin, spleen

## Abstract

Immune surveillance of tissues is primarily carried out by dendritic cells (DCs), which act as sentinels for the adaptive immune system. To accomplish this task, DCs migrate from tissues to regional lymph nodes, or from blood‐exposed regions of the spleen to the white pulp, to prime T cell responses. DC migration is a tightly regulated process that occurs at both steady state and during inflammation, and is dependent on sensing a wide array of chemoattractant molecules. Migration involves dynamic cytoskeletal rearrangement after signaling from chemotactic receptors, followed by rapid chemotaxis to specific regions of lymphoid tissues along gradients of chemoattractant molecules. In this review, we explore how DCs regulate the process of migration at the level of activation and receptor expression, chemoattractant sensing, and signaling to induce cytoskeletal rearrangement. We discuss differences in how DC subsets migrate, including the different regions these subsets localize to within lymphoid tissues and how these differences impact T cell responses. We also examine DC migration in the context of diverse tissue environments, with a focus on barrier sites. This comparison contributes to a holistic understanding of the common ways DC migration is regulated, as well as key differences that contribute to divergent adaptive immune responses.

## 1 Introduction

Dendritic cells (DCs) are an essential population of immune cells that act as a bridge between the innate and adaptive immune systems. DCs accomplish this task by surveilling a wide variety of tissues and immune compartments throughout the body for pathogens, cancer, and damage. Upon encountering a stimulus that DCs recognize as dangerous, DCs seeded in peripheral tissue become activated and migrate from tissue to secondary lymphoid organs (SLOs), where naïve T cells typically reside. Once they reach SLOs, DCs can present antigen to naïve T cells and activate adaptive immune responses characterized by antigen specificity and long‐lived memory. The migratory capacity of DCs thus allows them to communicate inflammatory insults to lymphoid tissues, where T cell priming generally occurs.

Conventional DCs (cDCs) are a distinct hematopoietic lineage of leukocytes that originate from the bone marrow via common DC precursors (CDP) and critically rely on Flt3L for their differentiation, as opposed to other types of antigen‐presenting cells (APCs) such as monocytes and macrophages [1, 2]. Related cell types, including Langerhans cells and plasmacytoid DCs (pDCs), have distinct ontogeny and functions from cDCs and will not be covered in this review. cDCs can be ontologically categorized into cDC1s and cDC2s, which have themselves recently been further subdivided into Notch2‐dependent cDC2As and KLF4‐dependent cDC2Bs [3, 4, 5]. cDC1s are dependent on the transcription factors BATF3 and IRF8 for their differentiation, and can generally be identified by flow cytometry on the basis of their positive expression of the markers CD11c, MHC‐II, XCR1, and CLEC9A, regardless of their localization within the body [2]. cDC2s are dependent on the transcription factor IRF4, and can be identified across locations as positive for CD11c, MHC‐II, Sirpα, and CD11b, along with sub‐subsets that express other markers depending on development and activation [2]. The cDC1 and cDC2 subsets have broadly been implicated in activating distinct branches of the immune system; cDC1s are particularly efficient at cross‐presentation and activating CD8^+^ T cells, whereas cDC2s are better at priming CD4^+^ T cells [6]. However, these are not necessarily strict rules, as cDC1s are capable of activating CD4^+^ T helper 1 (T_H_1) cells, and cDC2s can cross‐present to CD8^+^ T cells. Additionally, cDC2s have been shown to further specialize in the types of CD4^+^ T cells they activate, providing an additional layer of complexity.

In addition to the cDC ontogeny, DCs can be further categorized as migratory or lymph node‐resident [6, 7]. DCs develop from CDPs into pre‐DCs, which circulate the blood and, upon sensing local chemoattractants, seed various niches throughout the body to provide immune surveillance. Resident DCs are DCs that home to lymph nodes (LNs) directly from blood, whereas migratory DCs are DCs that first seed tissues and navigate to LNs upon stimulation. Migratory cDC1s typically express the integrin CD103, whereas resident cDC1s express CD8αα. In contrast, resident cDC2s in some lymphoid tissue express CD4 [8]. Migratory cDC2s, however, are not as easily distinguished from resident cDC2s as both typically express the integrin CD11b. Additional analysis of tissue‐specific markers may help identify migratory cDC2s; for example, intestinal migratory cDC2s express both CD103 and CD11b [9], whereas a subset of dermal [10] and lung [11] cDC2s express CD301b. The limitation of this, however, is that given the heterogeneity of cDC2s, these may actually be marking different subsets based on the cDC2A/B categorization; indeed, Notch2‐dependent cDC2As have been shown to express CD103 in the intestine [12], and Notch‐independent (KLF4‐dependent) cDC2Bs express CD301b in the lung [5, 13]. Future studies on the newly defined cDC2A and cDC2B subsets should further clarify whether these or other markers are broadly applicable to migratory and resident cDC2 identification, particularly using transcription factors such as T‐bet (cDC2A^+^, cDC2B^−^) [3].

It is important to note that all DCs are capable of the cellular process of migration, and indeed, even resident DCs migrate to different regions within the SLO [14, 15, 16]. Thus, the migratory/resident dichotomy is more descriptive of the initial location of DC seeding rather than their ability to migrate. Regardless, all cDC subsets migrate in order to carry out their surveillance function, and understanding how this complex process is regulated is critical to targeting distinct branches of the immune response [7].

## 2 Basic Requirements for Dendritic Cell Migration

### 2.1 DC Activation and Effects on Migration

The life of a migratory DC can be roughly broken up into three phases: (1) seeding and patrol of peripheral tissues, (2) activation and migration through tissue towards SLOs, and (3) positioning within lymphoid organs and activation of T cells. Each of these phases is regulated and guided by both internal and external signals, as the initiation of adaptive immunity must be done accurately and appropriately to avoid disorders such as autoimmunity or allergy. Indeed, DCs have been implicated in immune responses that lead to these pathologies [17, 18].

Upon encounter with activation signals, DCs undergo a number of important changes in their function. Maturation induces a downregulation in endocytosis in preparation for migration, as much of the machinery and signaling used to regulate endocytosis is repurposed to coordinate chemotaxis [19, 20]. This also likely prevents uptake and presentation of self‐antigens after DC activation, which would have the potential to result in autoimmunity. Additionally, DCs become less adherent to the extracellular matrix by decreasing the number of podosomes, or actin‐rich attachment structures, that they use to adhere to substrates [21, 22]. This results in the ability of DCs to migrate more rapidly toward chemoattractant gradients, additionally exemplified by their redistribution of the actin cytoskeleton and greater usage of the GTPase RhoA to regulate the speed of chemotaxis [20]. Cytoskeletal organization during migration will be covered in depth in Section 2.4, but it is clear that maturation sets up DCs to effectively and rapidly bring acquired antigens to T cells for presentation.

In addition to more immediate signaling and cytoskeletal changes upon maturation, key transcription factors are engaged to change the function of DCs. One of the most critical changes that dictate DC migration is the transcriptional downregulation of chemokine receptors such as CCR1 and CCR5 that guide DCs to tissues and prevent their exit, in parallel with the upregulation of the chemokine receptors CCR7, CCR8, and CXCR5, all of which guide DCs into SLOs or specific regions within SLOs under various conditions [23]. This induced change in chemokine receptor expression is essential in regulating DC migration and T cell activation; in particular, CCR7 is required for mature DC trafficking to SLOs, as the knockout of CCR7 abolishes DC migration to LNs across the body and within the spleen and hampers T cell responses significantly [8, 24, 25]. The expression of CCR7 is regulated by the transcription factor NFκB, which activates another transcription factor AP‐1 to induce CCR7 gene expression [26].

A number of extracellular stimuli regulate NFκB activation and CCR7 upregulation, highlighting an important convergence of signals by DCs to trigger migration to SLOs. Many inflammatory molecules have been demonstrated to directly activate DC maturation and CCR7 upregulation, including TNFα and IL‐1 [27]. A variety of pathogen‐associated molecular patterns (PAMPs) such as lipopolysaccharide (LPS) and polyI:C are sensed by DCs through pattern recognition receptors, including toll‐like receptors (TLRs), and induce CCR7 expression via NFκB [24, 28]. Damage‐associated molecular patterns (DAMPs) and alarmins can also induce CCR7 upregulation in a similar manner [29]. Additionally, several lipid signaling molecules have been shown to activate DC migration via CCR7. The fatty acid arachidonic acid (AA) is metabolized by cytosolic phospholipase A_2_ (cPLA_2_) into two branches of lipids: leukotrienes (LT) and prostaglandins (PG), both of which are signaling mediators implicated in innate immune activation and type 2 inflammation [30, 31, 32]. Human monocyte‐derived DCs (moDCs) chemotax towards gradients of LTB_4_ [33], and both LTB_4_ and LTC_4_ activate DC migration by inducing CCR7 surface expression [34, 35, 36]. Similarly, PGE_2_ activates NFκB‐induced upregulation of CCR7 [37, 38, 39], whereas its precursor PGD_2_ inhibits CCR7 upregulation [40, 41, 42]. Thus, DCs integrate environmental cues via a common pathway to reduce retention signals that keep them in tissues and induce migration to lymphoid organs.

Although the NFκB‐mediated induction of CCR7 is typically studied in the context of inflammation, it has also been implicated in steady‐state migration to lymphoid organs. In the skin, DCs traffic to LNs under homeostatic conditions, implying a steady‐state surveillance of tissue antigens [25, 39, 43]. This migration is dependent on NFκB, as deletion of the upstream regulator IKKβ leads to abrogation of DC migration and lower CCR7 expression, whereas deletion of MyD88 and Trif, signaling transducers of TLR and IL‐1 receptors [28], as well as deletion of TNF receptors, has no effect on steady‐state DC migration [43]. The mechanism by which NFκB is activated in migratory DCs in the absence of inflammatory conditions has recently been uncovered. DCs utilize a nuclear shape‐sensing mechanism when migrating through confined environments to translocate cPLA_2_ to the nucleus and produce PGE_2_, which then signals to activate NFκB and upregulate CCR7 [39]. Homeostatic DC migration has been linked to peripheral T regulatory (pTreg) cell induction [44, 45, 46, 47] and protection from autoimmunity [43], underlying the importance of migratory DCs even in the absence of external pathogens or inflammation.

### 2.2 Chemoattractant Sensing

In order for DCs to migrate to appropriate locations, either in tissue or lymphoid organs, they must follow guidance cues to navigate accurately. These most often are in the form of chemokines, which are small peptide signaling molecules that can be grouped into CC, CXC, CX_3_C, or XC families on the basis of the cysteine configuration at the N‐terminus [48]. However, other signaling molecules can act as chemoattractants for DCs, such as leukotrienes, sphingolipids, and oxysterols. Regardless of the specific molecule used, the principle for directing DC navigation is similar; chemoattractants form gradients within tissues and lymphoid organs, which are then sensed by DCs. Sensing of chemoattractants by surface receptors, typically G‐protein coupled receptors (GPCRs), triggers downstream signaling responses that direct cytoskeletal rearrangement and migration towards the gradient [49].

The cellular process of migration is initiated following the binding of a chemoattractant to a surface receptor, which triggers immediate signaling events to remodel the cell architecture for migration. Chemokine receptors are members of a large family of GPCRs, which consist of a transmembrane protein with an extracellular chemokine binding domain, and intracellular domains that bind to G proteins to transduce signaling [48, 50, 51, 52]. Typically, the G protein subunit Gα and the subunits Gβγ activate overlapping and distinct signaling modules to mediate downstream effector functions (reviewed by [53]). Although DCs express and utilize a wide array of receptors to sense chemoattractant molecules and trigger migration [23], by far the most well‐studied signaling pathway for DCs is the CCL19‐ and CCL21‐CCR7 axis. As a result, this section will primarily focus on the signaling events following CCR7 stimulation and how they regulate DC migration.

After acquisition of CCR7 expression, DCs migrate to LNs following gradients of both CCL19 and CCL21 [54]. CCL19 is a soluble chemokine that is produced by fibroblastic reticular cells in the T cell zones of LNs [55], as well as by DCs in humans [56]. CCL21 is also produced by reticular cells in LNs [55], as well as by high endothelial venules (HEVs) in mice but not humans [57]. HEVs are blood vessels that allow lymphocyte entry into LNs from the blood. Finally, lymphatic vessels are a primary source of CCL21 secretion, forming gradients of chemokine both in lymphatic vessels and in nearby tissue [58, 59]. CCL21 contains an elongated C‐terminal tail, allowing it to bind to proteoglycans [60], resulting in the fixation of CCL21 to cell and matrix surfaces and the creation of an immobilized chemokine gradient leading from tissue to lymphatics [59]. A recent study demonstrates that, although CCL21 is downregulated in lymphatics during an inflammatory response, CCL19 expression is maintained and cooperates with oxysterol‐EBI2 signaling to recruit lymphocytes into inflamed LNs, demonstrating that CCL19 and CCL21 have unique roles during different phases of immune responses [61].

Although DC migration is activated by both CCL19 and CCL21, the immediate events and cellular responses following binding to each cytokine differ slightly. Both chemokines induce increased endocytosis of exogenous molecules such as FITC‐dextran [62], yet CCL19 induces greater internalization of CCR7 than CCL21, indicating a selectivity in CCR7 endocytosis by CCL19 that is not applicable for other molecules [63]. CCL19‐mediated endocytosis of CCR7 is dependent on Arrestin 3 (also known as β‐arrestin 2) in the context of several immortalized cell lines [64], and upon internalization, CCL19 is removed from the receptor and targeted for degradation while CCR7 recycles back to the plasma membrane [63, 65]. This pathway results in a continued ability to sense chemokine by recycling CCR7, whereas soluble CCL19 is soaked up by migrating DCs. The soaking effect allows DCs to guide following cells by creating CCL19 gradients where more chemokine remains at the front of a group of migrating cells, facilitating collective migration to lymphatics [66].

The lack of CCR7 endocytosis in response to CCL21 likely evolved because of the fact that CCL21, unlike CCL19, is immobilized to proteoglycans on extracellular matrix proteins in tissues and lymphatic vessels, creating an endogenous gradient guiding DCs towards secondary lymphoid organs [59, 67]. Interestingly, when DCs are placed in the middle of equal but opposing gradients of CCL19 and CCL21, DCs preferentially migrate towards CCL21 despite similar binding affinities of CCR7 for each chemokine [68]. This may reflect the dispensable role of CCL19 for migratory DC function in vivo, as DCs are able to effectively migrate to LNs and prime T cells in the absence of CCL19 [69]. Despite the differential response of DCs to CCL19 and CCL21, the underlying mechanisms governing these mechanisms are still poorly understood.

In addition to CCL19 and CCL21 guidance into LNs, the signaling lipid sphingosine‐1‐phosphate (S1P) is expressed within the LN and spleen and cooperates with CCL19/21 signaling to guide lymphoid positioning of DCs [70, 71, 72, 73, 74]. cDCs express the S1P receptors S1PR1 and S1PR3, and have been shown both in vitro and in vivo to migrate toward S1P gradients [70, 75]. Although S1P is typically thought to regulate lymphocyte egress from the LN into the blood, blocking of S1P signaling in DCs by treatment with the receptor agonist FTY720 has been shown in several studies to block DC trafficking to LNs [76, 77, 78, 79], although whether the mechanism by which S1P recruits DCs to LNs is direct or indirect remains unclear. It is likely that the requirement for S1P signaling for guidance to LNs is dependent on the specific inflammatory environment and other local cues [31].

### 2.3 Signaling Events Downstream of Chemoattractant Sensing

Following binding by CCL19 or CCL21, several downstream signaling modules are rapidly activated in parallel to modulate DC migration and survival [53]. The phosphoinositide 3‐kinase (PI3K)/AKT signaling is the primary signaling cascade promoting DC survival after chemokine signaling [53]. PI3K is a kinase activated by GPCR signaling (such as CCR7) and phosphorylates PI(4,5)P_2_ to produce PI(3,4,5)P_3_. The production of PI(3,4,5)P_3_ regulates a variety of targets, including the recruitment of the kinase AKT and guanine exchange factors (GEFs) that activate Rho‐family GTPases to stimulate actin polymerization. Several studies have examined the role of the PI3K pathway in DC migration, with conflicting results [80, 81, 82]. Deletion of PI3Kγ inhibited the ability of immature DCs to migrate toward CCL3 and CCL5, and of mature DCs to migrate towards CCL19 [83]. However, in contrast to this study using murine PI3Kγ^−/−^ bone marrow‐derived DCs, human moDCs demonstrated no impairment in migration to CCL19 and CCL21 in the presence of a PI3K inhibitor [81]. Thus, the role of PI3K signaling in the direct regulation of migration within DCs remains to be determined. Despite this, a role for the PI3K pathway in regulating DC apoptosis downstream of CCR7 has been described. After stimulation with CCL19/21, AKT1 is activated and signals through NFκB to promote survival, as inhibition of PI3K reduces AKT1 activation and induces apoptosis in an NFκB‐dependent manner [80]. Future studies should attempt to clarify the role of PI3K signaling in the direct regulation of DC migration independently of its role in survival.

Multiple integrated signaling cascades orchestrate DC migration. One essential pathway in DC migration is the mitogen‐activated protein kinase (MAPK) pathway, including members ERK1/2, p38, and JNK [80, 81, 84]. MAPK signaling is characterized by a cascade of kinases that rapidly phosphorylate and activate downstream mediators; the cellular pathways targeted by these cascades are diverse and include cytoskeletal regulation, activation of transcription factors, modulation of metabolism, and many others. MAPK signaling is typically shown to be activated by Rho‐family GTPases such as Ras and Raf downstream of surface receptors, but this has not been formally demonstrated in DCs in the context of chemokine receptor signaling. However, the role of MAPK signaling has been demonstrated to impact DC migration, as inhibition of several MAPK members results in reduced numbers of mature DCs that migrate toward chemokine, though the speed of chemotaxis for cells that do migrate is unchanged [81]. The signaling of MAPK members is independent of PI3K/AKT signaling [81], and MAPK inhibition does not affect DC survival [80]. JNK phosphorylation in DCs is downstream of both ERK1/2 and p38 [81], and the inhibition of JNK signaling in the murine DC cell line BC1 reduced the number of migrated cells but not their CCL19‐induced endocytosis [84]. Despite the importance of this pathway in DC chemotaxis, the downstream targets of MAPK that regulate migration are still not well understood.

### 2.4 Cytoskeletal Organization During DC Migration

Cell migration through tissue requires a coordinated balance of forces that allows DCs to navigate towards chemokine gradients while maintaining cellular coherence and nuclear integrity, the loss of which results in nuclear rupture and cell death. To accomplish this, DCs dynamically rearrange their cytoskeleton during migration to create a polar cell with a distinct front and back, generate protrusive forces, contract the rear of the cell, and protect the nucleus from rupture [85]. Much of the study on cytoskeletal organization was historically conducted using immortalized fibroblast cell lines, which use a distinct mechanism of migration (termed mesenchymal). Mesenchymal migration is characterized by the extensive use of integrins to create focal adhesions, allowing the cell to pull itself forward using the extracellular matrix as a series of anchor points [85]. In contrast, DCs and other leukocytes use a more rapid amoeboid mechanism of migration characterized by the dispensable use of integrins, instead using actin‐based protrusions and myosin contractility to rapidly push and squeeze the cell body through confined spaces [85].

DCs have been shown to migrate independently of integrins both in vivo and in complex 3D collagen gels in vitro [86]. Although integrins are still required for DC attachment to 2D extracellular matrix (ECM)‐coated surfaces and for extravasation from the bloodstream into tissue, migration through tissue or collagen gels instead relies on actin‐mediated leading edge protrusions and myosin‐mediated contractility of the back of the cell [86]. DCs can also rapidly switch between using integrin‐based attachments on protein‐coated surfaces and amoeboid flowing on polyethylene glycol‐coated surfaces that lack attachment points for integrins [87]. Moreover, although fibroblasts prioritize migrating along paths of ECM even when these paths are inefficient at reaching the highest density of chemokine, DCs can prioritize more rapid migration by traveling on a direct path toward chemokine, switching between integrin‐dependent and ‐independent modes of migration [87]. Thus, DCs are more flexible in their ability to reach a desired location by not relying on attachment points to generate force for chemotaxis.

**FIGURE 1:**
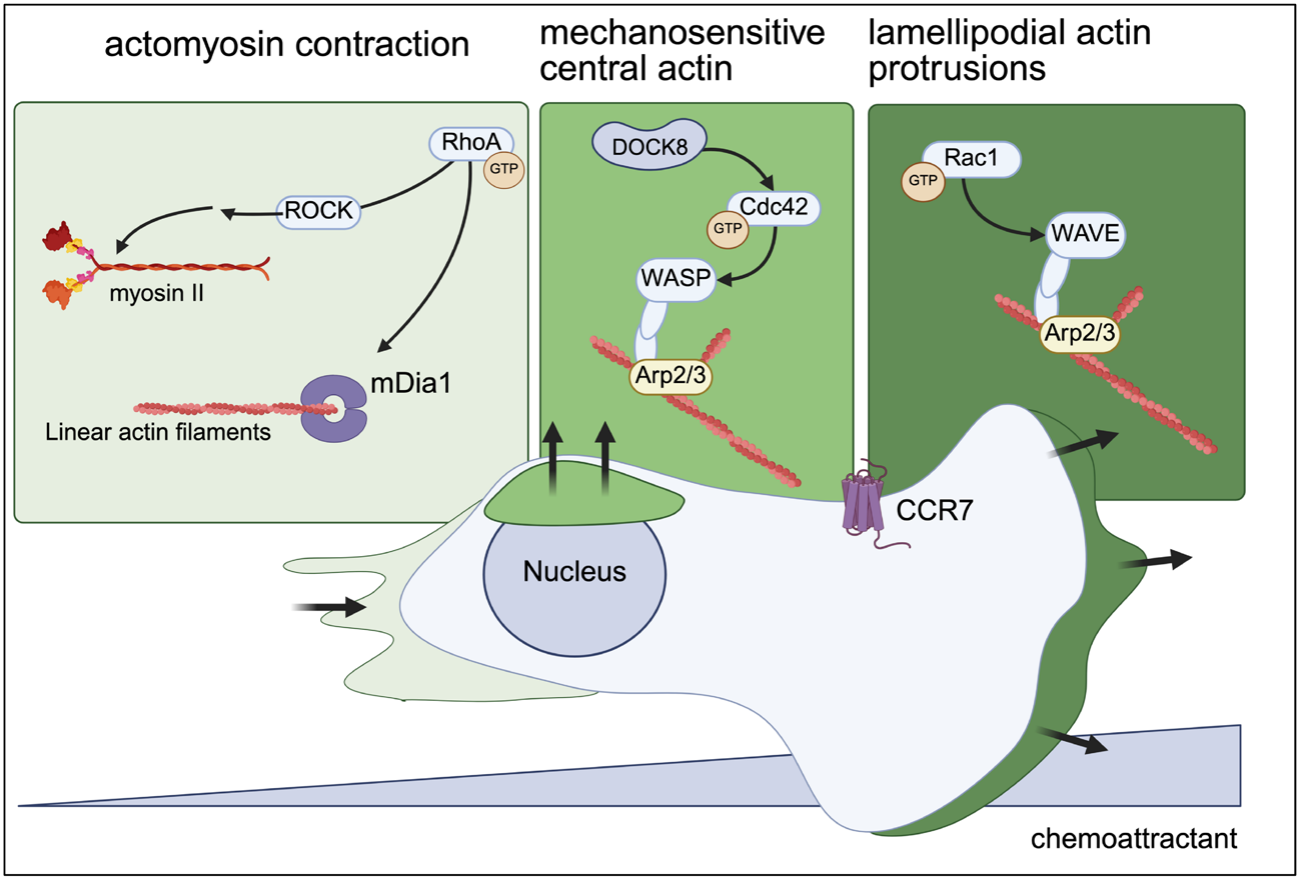
Spatial and functional specialization of actin polymerization by Rho‐family GTPases during DC migration. During DC migration, signal transduction downstream of CCR7 results in the activation of three actin polymerization pathways in distinct regions of the cell. These pathways are controlled by the Rho‐family GTPases Rac1, Cdc42, and RhoA. Rac1 activates the actin nucleating protein WAVE2 at the leading‐edge of the cell, which then activates Arp2/3 and actin branching. Rac1‐controlled leading‐edge actin is primarily responsible for lamellipodial protrusions and forward propulsion. At the back of the cell, RhoA signals to polymerize linear actin filaments by activating the nucleating protein mDia1. RhoA also activates several kinases such as ROCK, which further signal to activate myosin II. Thus, the action of RhoA results in the formation of linear actin‐myosin complexes, which contract the back of the cell and determine the migratory speed of the cell. Finally, the GTPase Cdc42, which is regulated by the GEF DOCK8, activates WASp to nucleate branched actin structures vertically over the nucleus of migrating cells. This actin pool is responsive to external mechanical pressure and is critical in protecting the nucleus while the DC moves through confined spaces. Together, these actin pathways synergistically regulate DC motility by maintaining a balance between forward protrusions, migratory speed, and cellular integrity. This figure was created using BioRender (https://BioRender.com/liqimvs).

Actin polymerization pathways are primarily controlled by Rho‐family GTPases, namely, Rac1, Cdc42, and RhoA [88, 89, 90, 91], all of which are activated in DCs upon chemokine sensing [62, 81] (Figure 1). These GTPases are critical signaling mediators that are active when bound to GTP but not GDP, and are thus directly regulated positively by the activity of guanine nucleotide exchange factors (GEFs) and negatively by GTPase‐activating proteins (GAPs). Rho‐family GTPases signal downstream to a variety of mediators, including formins such as mDia1 that nucleate linear actin filaments [92], Wiskott‐Aldrich syndrome protein (WASp)‐family proteins that activate Arp2/3 to nucleate branched actin sheets [93], and various kinases that tune the activity of both actin‐nucleating proteins and myosin‐activating proteins.

Rho‐family GTPases are generally thought to occupy specific subcellular niches in the coordination of the actin cytoskeleton. Rac1 localizes primarily to the leading edge of a polarized cell and mediates branched actin polymerization to form lamellipodia to generate forward protrusive forces; Rac1 activates WASp family Verprolin‐homologous protein‐2 (WAVE2), which nucleates actin polymerization at the leading edge. In the context of DCs, Rac1 has been shown to be essential for dendrite formation [94, 95, 96], leading‐edge lamellipodial actin protrusions [97], and successful migration [95]. Similarly, WAVE complex members have been imaged localizing to the leading edge of migrating DCs, and the deletion of WAVE complex members abolishes lamellipodia and leading‐edge pathfinding to the detriment of migration [98, 99].

While Rac1 and the WAVE complex localize to the leading edge of migrating cells, RhoA typically localizes to the rear to stimulate actomyosin contraction. Indeed, in DCs where RhoA is inhibited or knocked out, F‐actin polymerization at the rear of polarized DCs is decreased [20]. Furthermore, inhibition or deletion of the formin mDia1 or the kinase ROCK, both of which are regulated by RhoA, results in slower chemotaxis, poor directionality, and overall decreased DC migration [20, 100, 101, 102, 103]. Finally, inhibition of myosin II activity results in poor DC migration, characterized by long cell bodies because of poor cell body retraction during migration, particularly in very complex or confined environments [86, 104].

In contrast to Rac1 and RhoA, which typically have defined roles in the front and rear of a polarized cell, respectively, the role of Cdc42 is more cryptic. Cdc42 is a Rho‐GTPase that is broadly involved in establishing cell polarity and maintaining directionality during migration towards a chemokine [105]. Cdc42‐deficient DCs have overall intact rates of actin polymerization, but develop multiple leading edges that attempt to pull the cell in multiple directions, thus breaking polarity and leading to cell entanglement and shearing in fibrillar networks [106]. The role of Cdc42 has subsequently been further elucidated through studies on the protein dedicator of cytokinesis 8 (DOCK8). DOCK8 is an atypical GEF that has been shown to specifically bind and activate Cdc42, but not RhoA or Rac1, via its DOCK homology region (DHR)‐2 domain [107]. Loss of DOCK8 from DCs phenocopies the migratory defect from loss of Cdc42, producing multipolar cells that can migrate in simple environments but not in 3D matrices or in vivo [107, 108]. Interestingly, when DOCK8 is deleted from DCs in vivo, the migratory deficiency is restricted to cDC2s but not cDC1s, raising important implications that different DC subsets use distinct mechanisms to regulate their migration [109].

Recent studies have provided important mechanistic insights as to how DOCK8 and Cdc42 regulate DC polarity during migration. In both T cells [110] and DCs [111], DOCK8 is essential for a pool of central F‐actin that is activated in response to mechanical pressure. When cells migrate through confined environments, DOCK8 localizes to the center of the cell and activates this central actin pool to push against the external matrix and preserve nuclear integrity. Loss of DOCK8 results in relocalization of F‐actin to the leading edge, resulting in poor migration and cell entanglement in the ECM [111]. The regulation of this central pool of actin is also shown to be in part dependent on Cdc42 and its downstream actin nucleator WASp [99, 111], which has been previously shown to be important for DC migration [112, 113, 114, 115, 116, 117]. The central actin pool seems to be in a regulatory balance with the leading‐edge actin, as an anti‐correlation was observed between these two pools of actin [111]. These findings together create a model by which DOCK8 and Cdc42 regulate polarity: when DCs encounter an obstacle to nuclear passage, leading edge F‐actin is relocalized to the central actin pool to displace the ECM and limit additional protrusions, allowing the nucleus to pass and maintain cellular coherence [111].

The coordination of several signaling pathways downstream of chemokine sensing results in a sophisticated control of the cytoskeleton during DC migration (Figure 1). Rac1‐mediated forward protrusions and RhoA‐mediated rear contraction dictate the locomotive speed of migrating DCs, whereas Cdc42 acts as a braking mechanism when the cell is forced through constrictions to maintain nuclear integrity. These mechanisms together allow DCs to make their way through dense ECMs in the tissue to their eventual destinations in SLOs, where they can prime T cell responses. We will now discuss the specific migratory cues and mechanisms that guide DC subset migration in a variety of tissues and SLOs, as well as the resulting T cell responses.

## 3 Splenic DC Migration

### 3.1 DC Subsets Within the Spleen

The spleen is a major site of immune surveillance, functioning to protect the body from systemic immune insults such as bloodborne pathogens or cancers [118]. The spleen can be roughly divided into the red pulp (RP) and white pulp (WP), which are separated by a marginal zone (MZ) in mice and a perifollicular zone in humans. The RP is a blood‐exposed region where red blood cells (RBCs) and antigens flow through the spleen. In the RP, aging RBCs are removed from circulation, and immune cells survey the blood for pathogens or DAMP signals. The WP contains most of the immune cells in the spleen, and can be thought of as a series of LN‐like structures with T cell zones, B cell follicles, and germinal centers. The WP is not blood‐exposed despite lacking a capsule like lymph nodes. Anything larger than 60 kDa cannot enter the WP without carriage from a cell [8, 119, 120, 121], underlying the importance of cell migration for proper surveillance of circulating blood by the spleen.

DCs are critical for T cell responses originating in the spleen. Most cDCs in the spleen can be divided into three subsets: cDC1s are CD8α^+^ and XCR1^+^, and variably express DEC‐205, CD103, and CD207/langerin (in Balb/c mice [122]); cDC2As are Sirpα^+^, CD11b^+^, CD4^+^, Esam^+^, and T‐bet^+^; cDC2Bs are Sirpα^+^, CD11b^+^, CLEC12A^+^, CX_3_CR1^+^, CD301b (Mgl2)^+^, PD‐L2^+^, and T‐bet^−^ [3, 4, 5]. Both cDC2As and cDC2Bs in the spleen express the classical cDC2 marker DCIR2 (also known as CLEC4A4), which is recognized by the monoclonal antibody 33D1, but its expression is higher in Esam^+^ cDC2As [12]. In contrast, CD11b is expressed at higher levels in cDC2Bs [12]. The ontogeny of these cDC2 subsets has been elucidated over the years, with recent studies defining distinct pre‐cDC2 populations that give rise to cDC2A/B cells [5, 123, 124]. Additionally, they require different signaling pathways and transcriptional regulators for their development, with cDC2As dependent on Notch2 and RBPJ [12, 125], retinoic acid (RA) signaling [126], LTβ signaling [121, 127, 128, 129, 130], and TCF4 [124]. cDC2Bs are dependent on KLF4 [5, 13, 123, 124], and both cDC2 subsets are mostly dependent on IRF4 [12, 131].

Despite the recent work on cDC2 ontogeny, the heterogeneity of this population of DCs and their similarity to related cell types pose a challenge for identification, especially when assessing cDC2 migration and function in older studies. A recently identified transitional DC (tDC) population shares a lymphoid lineage with plasmacytoid DCs (pDCs) and can transition into Esam^+^ cells resembling cDC2As [132, 133]. However, several ontogeny studies have argued that tDCs are a pre‐DC2 subset dependent on TCF4 [123, 124], a transcription factor shared with pDC development [134]. An additional wrinkle of complexity for cDC2 identification was added by the discovery of a DC3 subset, which arises from a Ly6C^+^ monocyte‐dendritic cell progenitor, in contrast to cDCs which arise from a CDP [3, 135, 136, 137, 138]. DC3s have overlapping expression of several markers with cDC2Bs in particular, such as Sirpα and CD301b, but can be distinguished in some studies by their expression of CD16/32 (FcγRIII/RII) [139]. The role of DC3s in splenic immune responses remains to be elucidated, and thus these cells will not be extensively discussed in this review. In contrast, the other DC subsets have clear roles in initiating adaptive immunity in the spleen. Given the complexity of cDC2 differentiation and identification, we will largely refer to them on the basis of the markers used in a given study rather than as a specific sub‐subset.

### 3.2 Strategic Positioning of DCs Within the Spleen

**FIGURE 2:**
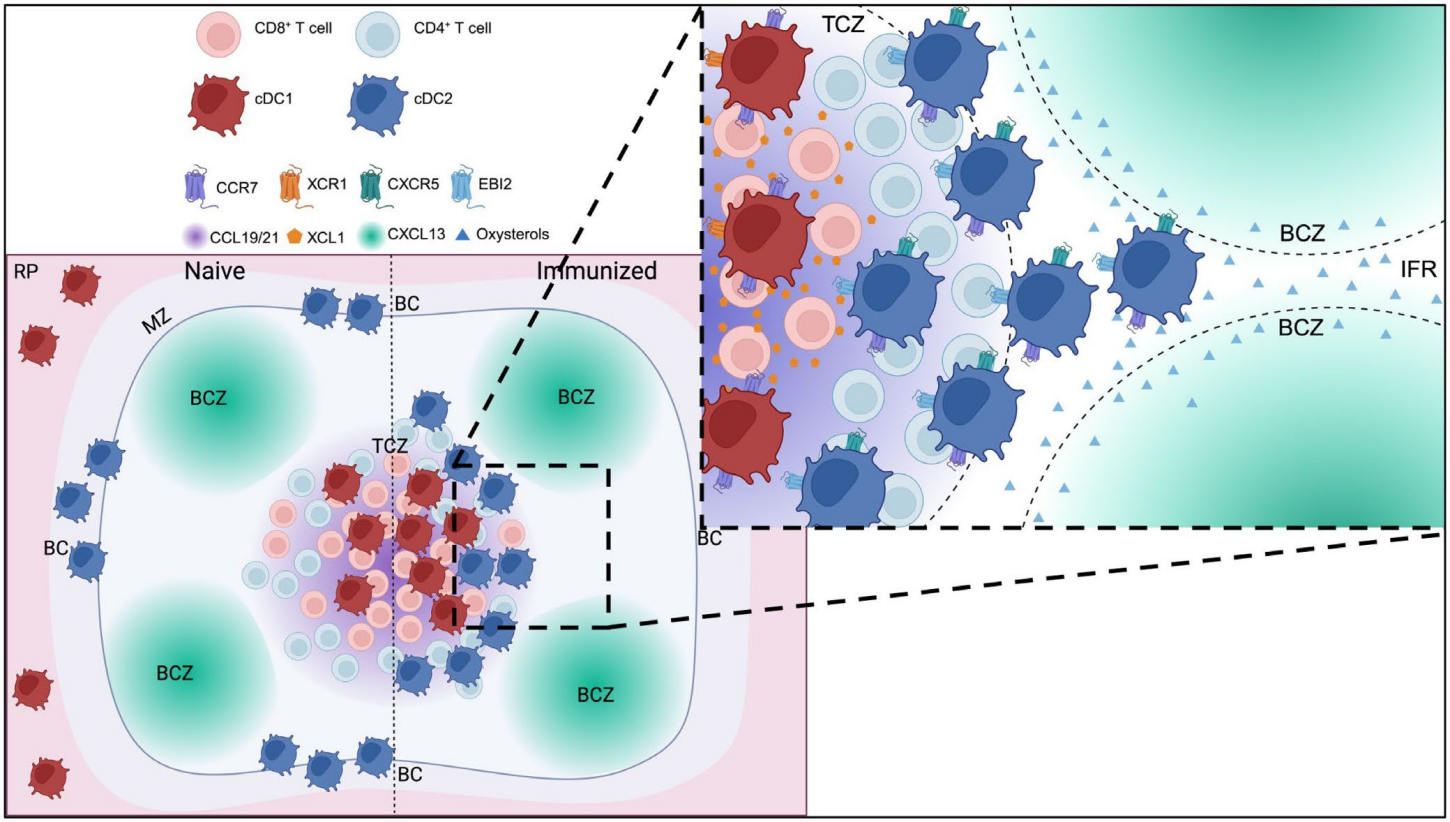
DC subset migration in the spleen is controlled by both cooperating and competing chemoattractant gradients. In a naive spleen, cDC2s primarily localize to bridging channels, while cDC1s can be found in both the RP and the T cell zone (TCZ). After immunization, cDC1s migrate to the deep TCZ and cDC2s migrate to the outer TCZ and interfollicular region (IFR). cDC1 migration is primarily dictated by higher expression of CCR7 relative to cDC2s, as well as their characteristic expression of XCR1. CD8^+^ T cells produce the chemokine XCL1, fostering close interactions between cDC1s to promote efficient cross‐presentation. cDC2s express CCR7 to home to the T cell zone, but additional expression of the receptors CXCR5 and EBI2 results in a different localization than cDC1s. CXCR5 recognizes CXCL13, a chemokine primarily produced in the B cell zone (BCZ), and EBI2 recognizes oxysterols produced by stromal cells at the BCZ border and IFR. The expression of receptors that recognize competing chemoattractant gradients results in cDC2 localization primarily at the T‐B border, which is likely advantageous for promoting interactions with CD4^+^ T cells and leading to eventual humoral responses via cooperation between CD4^+^ T cells and B cells. As a result of this, we propose that in addition to previously observed antigen‐processing differences between cDC1s and cDC2, their preference for priming CD8^+^ and CD4^+^ T cell responses, respectively, is due to sensing and migration to several distinct chemoattractant gradients. This figure was created using BioRender (https://BioRender.com/x3uv25k).

To survey the blood, migratory DCs are localized in various regions of the RP and MZ, and their homeostasis and distribution is strictly regulated [118, 140] (Figure 2). At steady state, CD8αα^+^ DEC‐205^+^ resident cDC1s localize mostly to the T cell zone of the WP [141], whereas a migratory subset of cDC1s (described in various studies as XCR1^+^, CD103^+^, or langerin^+^) resides in the RP [8, 122, 142]. In contrast, most cDC2s localize to the bridging channel (BC) [8, 71, 121, 143, 144], which acts as a conduit through which immune cells migrate between the RP and the T cell zone of the WP. S1P signaling via the S1PR1 and S1PR3 receptors has been shown to control DCIR2^+^ cDC2 localization to the BC, and inhibition via FTY720 treatment caused cDC2 redistribution throughout the MZ in the spleen [71], but this was shown to be an indirect effect because of displacement of MZ B cells rather than signaling of S1P on cDC2s [145]. Additionally, oxysterols play a critical role in localizing cDC2s to the BC. cDC2s but not cDC1s express the receptor EBI2 [121, 144], which recognizes the oxysterol ligands 7α,25‐HC and 7α,27‐HC. 7α,25‐HC gradients are created in the spleen by stromal cells in the outer region of B cell follicles and interfollicular regions [146], whereas 7α,27‐HC production is enriched in BCs. Loss of the EBI2 receptor from DCs results in fewer splenic cDC2s and mislocalization of cDC2s to the RP or T cell zone [121, 144]. Additionally, loss of 7α,27‐HC production results in the specific depletion of cDC2s from the BC at steady‐state [147].

EBI2‐mediated positioning is also important for localizing cDC2s to areas that provide signals for proper survival and differentiation, as reduced numbers of cDC2s in EBI2‐deficient mice are rescued by providing additional activation of the LTβ receptor [121]. LT signaling is critical for cDC2 homeostasis in lymphoid organs [129, 130], and is normally provided by interaction with B cells that express ligands for LTβR [129]. B cells also use EBI2 for proper splenic positioning [146] and thus are brought to the proper location to promote cDC2 expansion in the spleen. Additionally, for cDC2As dependent on Notch2 signaling, splenic fibroblasts express the Notch2 ligand Delta‐like 1 (DL1) [148] and co‐localize with DCIR2^+^ DCs in what appears to be the MZ/BC [125], indicating that the positioning of cDC2s sets up proper cDC2A differentiation.

### 3.3 DC Migration to the WP and T Cell Activation

The migration of both cDC1s and cDC2s from the MZ/BC to the WP is a critical step in initiating both tolerance against self and inflammatory responses against pathogens. DC migration to the T cell zone of the WP is induced by a variety of activating stimuli, including LPS [71, 142, 149, 150, 151], Toxoplasma gondii [150], various whole bacteria [152, 153, 154, 155, 156], polyI:C [142], apoptotic cells [122], and transfused foreign RBCs [8, 121, 157, 158], among others. The common link between most is ligation of pattern‐recognition receptors (PRRs) on DCs to stimulate maturation and CCR7 upregulation. Indeed, migration of DCs upon maturation to splenic T cell zones has been shown to be dependent on CCR7 recognition of CCL19/21 [8, 121, 157, 159].

Despite the ability of both cDC1s and cDC2s in the spleen to migrate to T cell zones upon activation, they initiate different immune responses. Antigens specifically targeted to splenic cDC1s or cDC2s via intravenous anti‐DEC‐205 and anti‐DCIR2 antibodies, respectively, resulted in the preferential activation of CD8^+^ T cells by cDC1s and CD4^+^ T cells by cDC2s [143]. This was hypothesized to be due in part to more efficient formation of peptide:MHC‐II complexes by cDC2s and the specialization of cDC1s to cross‐present exogenous antigen to CD8^+^ T cells [160]. However, a similar study using the same antibody targeting strategy but the strong type 1 adjuvant polyI:C observed superior T_H_1 priming by cDC1s compared to cDC2s [161], consistent with previous reports that migratory splenic cDC1s produce IL‐12, which is critically important for T_H_1 differentiation [150, 162, 163]. Thus, the previous dogma that migratory cDC1s are limited to CD8^+^ T cell priming is an oversimplification. However, there is a clear preference for particular DC subsets to initiate particular types of T cell responses. Our group has proposed that one reason for this selective T cell priming relates to the preferential migration of cDC1s to the CD8^+^ T cell region of the splenic T cell zone, whereas cDC2s co‐localize with CD4^+^ T cells at the periphery of the T cell zone [8] (Figure 2). Beyond this co‐localization, there are further specializations in terms of the nature of the T cell response initiated within the spleen by DC subsets, which we will review next.

Splenic cDC1s are particularly suited to differentiate Foxp3^+^ Treg cells from naïve CD4^+^ T cells because of their ability to produce TGFβ, whereas cDC2s cannot produce TGFβ but can differentiate Tregs if exogenous TGFβ is supplied [164]. Migratory CD103^+^ langerin^+^ cDC1s are particularly efficient at apoptotic cell uptake in the marginal zone compared to CD103^−^ cDC1s, resulting in migration to T cell zones and presentation of cell‐associated antigens [122]. The authors further demonstrated these results in tolerance to cell‐associated antigens, and that selective depletion of migratory cDC1s results in a loss of tolerance, presumably via the reduced ability to produce Tregs against antigen, though this was not specifically demonstrated. cDC1s lacking MHC‐II, and thus unable to prime CD4^+^ Tregs, are unable to impart tolerance and dampen CD8^+^ T cell‐mediated activation [165, 166].

As opposed to cDC1s, splenic migratory cDC2s have been demonstrated in various studies to induce T follicular helper (T_FH_) differentiation and antibody responses, in particular Notch2‐dependent cDC2As [167]. This has been demonstrated to be due in part to intrinsic characteristics of cDC2s, such as higher expression of OX40L and ICOSL [168], which are critical ligands for initiating T_FH_ development. In addition to expression of key surface proteins, cDC2 migration and positioning in the spleen are key factors in their ability to induce T_FH_ cells and humoral responses. We and others have shown that cDC2s migrate from the bridging channel to the outer region of the T cell zone, which is enriched for CD4^+^ T cells, upon immunization with foreign RBCs [8, 121]. Ablation of this migration with either *Ccr7* ^−/−^ or *Dock8* ^−/−^ mice results in defective CD4^+^ T cell proliferation [8]. Further, cDC2s are required for antibody responses to transfused RBCs [8, 120, 121, 144, 157, 167], underlying their preference for differentiating T cells that help B cell responses.

In addition to CCR7‐mediated migration to T cell zones, additional chemotactic factors are important for fine‐tuning cDC2 migration and positioning for T_FH_ induction. In addition to supporting cDC2 positioning at the BC at steady‐state, EBI2‐mediated migration is essential for cDC2‐mediated T cell and antibody responses [121, 144, 169]. Oxysterol gradients function to promote close interactions between activated CD25^+^ cDC2s and CD4^+^ pre‐T_FH_ cells by positioning both cells in an EBI2 and 7α,25‐HC‐dependent manner to the outer region of the T cell zone [147, 169], where cDC2s and pre‐T_FH_ cells have been shown to co‐localize [170]. Finally, CXCL13 is important for the migration of both T_FH_ cells and B cells into the germinal center light zone via the chemokine receptor CXCR5. A subset of DCs has been demonstrated to express CXCR5 and localize to B cell germinal centers [128], likely as part of mediating full T_FH_ development. We subsequently demonstrated that cDC2s express higher levels of CXCR5 than cDC1s in LNs [109], implicating locational positioning of migratory cDC2s in the spleen as a critical mediator of proper T_FH_ development and antibody production.

### 3.4 Hijacking of DC Migration by *Listeria*

Despite the previous discussion detailing the importance of splenic DC migration in proper adaptive immune responses, there are rare cases where DC migration is detrimental rather than beneficial. *Listeria monocytogenes* is an intracellular bacterium that enters the blood and is taken up in the spleen by macrophages in the MZ, which can then trans‐infect nearby cDC1s. Infection of cDC1s causes activation and migration, resulting in shuttling of *Listeria* to the T cell zone to establish a productive infection [171]. Migration by cDC1s is thus critical for *Listeria* infection and pathology [153, 154, 155], despite the necessity of cDC1‐mediated CD8^+^ T cell priming for *Listeria* clearance [171]. Using *Listeria* infection and *Dock8* ^−/−^ mice that lack both cDC2 migration and MZ B cells, we uncovered cross‐talk between multiple MZ cell types that regulates cDC1 migration into the T cell zone. In this model, IL‐10 from MZ B cells dampens lysosomal degradation of Listeria in macrophages, resulting in higher bacterial burden and greater capability of the bacteria to cross‐infect cDC1s in the MZ and trigger their migration [156]. Thus, Listeria takes advantage of MZ cellular communication to co‐opt cDC1 migration into the T cell zone and establish a niche for proliferation and pathogenesis.

Understanding factors that mediate splenic DC migration has important implications for not only harnessing beneficial migration to initiate effective adaptive immune responses, but also for inhibiting detrimental migration that initiates improper antibody responses against transfused RBCs or contributes to *Listeria* pathogenesis. Further, many of the principles of DC migration and localization observed within the spleen apply to DC migration and homing from different tissues to their respective draining LNs, as we will now review.

## 4 DC Positioning Within LNs

Once inside LNs, DCs are positioned by the competing pulls of multiple local chemoattractant gradients [7]. In addition to CCR7, cDC2s express CXCR5 and EBI2, which recognize CXCL13 and oxysterols respectively [109, 121, 147, 169, 172]. Both CXCL13 and oxysterols are produced largely within B cell zones, and the combined sensing of CCL19/21 in the T cell zones and CXCL13/oxysterols in the B cell zones results in migratory cDC2 localization to the T cell–B cell border zone of LNs [109, 172]. This positioning is particularly advantageous for activating T_FH_ cells [109, 173] as well as T_H_2 cells [172, 174].

In contrast, cDC1s are guided to T cell zones primarily by the expression of CCR7, where they can directly interact with CD8^+^ and CD4^+^ T cells [7, 175]. The higher CCR7 expression on cDC1s than cDC2s likely contributes to their stricter localization to T cell zones [109]; however, the expression of other chemokine receptors such as XCR1, which recognizes the chemokine XCL1 produced by CD8^+^ T cells [176, 177], also enforces their localization in the deep T cell zone. cDC1s also express enzymes that digest oxysterols, preventing B cells and cDC2s that express EBI2 from inappropriately moving into the T cell zone [147].

Production of chemokines such as CXCL10 by DCs in the T cell zone has been shown to guide CD4^+^ T cells into the T cell zone and promote T_H_1 differentiation, presumably through interactions with cDC1s [178]. Additional accessory cells also migrate to these regions and participate in guiding the evolving T cell response [16, 179]; such cellular concentrations have been called “spatial microenvironments” [16] that shape the differentiation of activated T cells on the basis of the nature of the inflammation [6].

Thus, local gradients position cDC subsets within LNs near the type of T cells they preferentially activate [7, 109, 175, 180, 181, 182], creating an efficient system by which migratory DCs bring antigen directly to the relevant responder [7].

## 5 Dermal DC Migration

### 5.1 Dermal DCs and Homeostatic Surveillance

The skin is an important barrier that provides primary protection from external pathogens and harmful stimuli. DCs, along with several other types of antigen‐presenting cells, reside in the skin and migrate to draining lymph nodes at both steady state and after activation. These APC subsets have diverse hematopoietic origins, consisting of monocyte‐derived DCs, macrophages, and cDCs [183]. The migratory capacity of one skin‐resident cell type in particular, called Langerhans cells (LCs), has been heavily studied over the years. LCs were originally thought to be a subset of DC specific to the skin epidermis on the basis of their morphology, co‐expression of surface markers such as langerin with some dermal DCs, and their capacity to migrate to skin‐draining LNs and activate T cells [184]. However, lineage tracing studies revealed that LCs are of embryonic origin [185, 186], and thus more closely related to macrophages rather than cDCs [1]. Therefore, LCs will not be a major focus of this section.

cDCs in the skin reside below the epidermis in the dermis and can be roughly divided into three subsets on the basis of historical use of markers. Migratory cDC1s are mostly CD103^+^, langerin^+^, XCR1^+^, and CLEC9A^+^, and migratory cDC2s are Sirpα^+^, and can be subdivided into a CD11b^+^ subset and a CD11b^−/lo^ “double negative” subset. Several studies have also demonstrated the presence of a CD301b^+^ PDL2^+^ cDC2 subset, which likely aligns with the cDC2B subset based on recent ontogeny work and may overlap with the CD11b^+^ and CD11b^−/lo^ dermal cDC2 subsets, depending on the study. Regardless, as discussed in the section on splenic DC migration, overlaying recent ontogeny work onto previous studies that have limited surface marker staining risks mischaracterizing cells; thus, we will again primarily discuss migratory cDC2s in the skin and other tissues on the basis of markers rather than cDC2A/B classification.

At steady state, migratory DCs in the skin engage in random motility to survey the tissue [187], followed by CCL21‐directed migration to draining LNs [188]. This homeostatic process relies on expression of CCR7 despite the lack of inflammatory stimuli that typically regulate DC maturation, and results in the appearance of “semi‐mature” DCs in draining LNs [25]. Loss of CCR7 results in a lack of migratory DCs in LNs, as well as a loss of antigen‐specific CD4^+^ T cell proliferation in LNs after immunization with both OVA alone or OVA with CpG, a strong type 1 adjuvant [25]. Homeostatic DC migration is governed by NFκB signaling; a DC‐specific deletion of IKKβ, an upstream regulator of NFκB, resulted in a near complete loss of migratory DCs from skin LNs, fewer Tregs in skin LNs, and spontaneous autoimmunity [43]. The activation of NFκB and subsequent upregulation of CCR7 is regulated by a nuclear shape‐sensing pathway that results in cPLA_2_ activation and PGE_2_ production [39]. Interestingly, skin CD11b^+^ cDC2s migrate more to LNs at steady‐state compared to cDC1s, and are more reliant on the cPLA_2_ pathway for migration than cDC1s [39]. Given that a loss of NFκB signaling results in almost complete loss of all migratory DCs from skin LNs [43], it seems likely that steady‐state cDC1 migration is regulated by a separate pathway that also activates NFκB, though this remains to be determined.

### 5.2 Kinetics of Dermal DC Subset Migration

Skin inflammation induces DC activation and CCR7‐ and S1P receptor‐dependent migration to skin‐draining LNs [76, 79] (see Section 2.2). The kinetics of DC migration from the skin have been measured by a variety of techniques, both at steady state and during inflammation. Injection of a fluorescent protein into the ears of mice, followed by staining with an antibody specific for peptide:MHCII complexes allowed detection of DCs that took up and presented antigen [189]. This study revealed that DCs migrating from the skin are detected in the lymph node about 24 h post‐injection, and that these DCs processed and presented antigen onto MHCII [189]. Most of the antigen^+^ peptide:MHC‐II^+^ migratory DCs are CD11b^+^, indicating that they are cDC2s, which was confirmed in a subsequent study from our lab using skin immunization [109] and another study using both skin irritants and tape‐stripping [190]. Skin irritation and fluorescein‐based tracking additionally demonstrated that CD11b + dermal DCs and langerin+ dermal DCs (likely cDC2s and cDC1s, respectively) peaked about 24 h after administration, whereas dermal DCs negative for both markers peaked a day later [191]. An elegant study using photoconversion to mark skin DCs demonstrated that migratory DCs reach the draining LN as early as 6 h after photoconversion [190], with migratory cDC2s reaching their peak numbers in the LN at 24 h post‐conversion and migratory cDC1s at 72 h. Thus, skin DC subsets have different migratory kinetics to draining LNs and are differentially triggered to migrate following skin damage.

### 5.3 Dermal DC Migration to Initiate Distinct T Cell Responses

Migratory dermal cDC1s contribute to the surveillance of skin‐associated antigens and the promotion of healthy barrier integrity. In several studies where OVA is expressed in keratinocytes of the skin, migratory cDC1s are critical for the cross‐presentation of cell‐associated OVA antigens to CD8^+^ T cells [192, 193]. The sampling of cell‐associated antigens is likely also important for tolerance in the skin, as the steady‐state NFκB‐dependent migration of cDC1s generates Tregs specific for keratinocyte‐derived OVA [45]. cDC1s also promotes healthy skin barrier environments by priming IL‐17 producing, epidermis‐homing CD8^+^ T cells against pathogenic bacterial colonization [194]. CD8^+^ T cell localization to the epidermis has also been shown to depend on latent TGFβ activation by migratory DCs expressing the αvβ8 integrin [195]. Finally, cDC1s survey the skin for pathogens, such as viruses and pathogenic bacteria. They also prime both CD8^+^ and T_H_1 responses against fungi such as *Candida albicans* [196]. cDC1s cross‐present viral antigens in herpes simplex virus (HSV) infections, though there is some discrepancy regarding whether migratory cDC1s directly prime CD8^+^ T cells [192], or hand off viral antigens to LN‐resident cDC1s for presentation [197]. Later work elegantly showed that migratory DCs transport viral antigens from the skin to prime CD4^+^ T cell help, followed by handing off antigen to LN‐resident cDC1s, which prime CD8^+^ T cells with CD4^+^ T cell help [182].

A large body of research has focused on the T_H_2‐priming capacity of cDC2s; in particular, the CD301b^+^ dermal cDC2 subset has been demonstrated to be particularly tailored to differentiate T_H_2 cells. CD301b^+^ cDC2s are dependent on the transcription factors KLF4 [13] and IRF4 [198] for their differentiation in the skin, and also require IRF4 for proper CCR7 expression and migration to lymph nodes [199]. In addition to CD301b, these cDC2s are characterized by expression of both PD‐L1 and PD‐L2 [198, 200, 201]. CD301b^+^ cDC2s have been shown to express CD11b [10], although a study demonstrating a role for homeostatic IL‐13 on the T_H_2 priming function of KLF4‐dependent cDC2s described the DCs as PD‐L2^+^ but CD11b^−/lo^, though they did not co‐stain for CD301b [202]. CD301b^+^ cDC2s migrate in response to skin inflammation and irritation, and are required for the differentiation of antigen‐specific T_H_2 cells in response to several type 2 stimuli, such as papain [198, 200, 203, 204, 205, 206], alum [200, 203, 206], FITC skin painting [10, 199, 207], the helminth parasite *Nippostrongylus brasiliensis* [198, 200, 203, 206], the allergen house dust mite (HDM) [13, 208], and the parasitic flatworm Schistosoma mansoni [13]. The particularly strong T_H_2 polarization by CD301b cDC2s has recently been attributed to their IL‐2 secretion and expression of CD25, a subunit of the IL‐2 receptor complex (IL‐2Rα) [206]. CD40 ligation on CD301b^+^ cDC2s induced the secretion of IL‐2 and surface expression of CD25, which the authors demonstrated directs IL‐2 towards cognate CD4^+^ T cells for T_H_2 induction [206]. The intrinsic ability of CD301b^+^ cDC2s to produce IL‐2 and express PD‐L1/PD‐L2 may also explain their antagonistic relationship with T_FH_ cells [201], the development of which has been shown to be negatively regulated by these signals [173, 209].

The migration of CD301b^+^ cDC2s after allergic sensitization is unusual, as type 2 inflammation does not strongly induce CCR7 on these DCs [203]. While the low baseline level of CCR7 expression is still required for their migration to the LN, CD301b^+^ cDC2s also express the chemokine receptor CCR8 and are dependent on the expression of the CCR8 ligand CCL8 in the LN after allergic inflammation for entry into the LN parenchyma [203]. Motility of CD301b^+^ cDC2s is additionally stimulated by pain‐sensing TRPV1^+^ neurons [204], which secrete substance P upon the sensing of allergic inflammation and IL‐3 by γδ T cells [210]. Once inside the LN parenchyma, CD301b^+^ cDC2s localize near the HEVs based on sensing of HEV‐derived S1P, allowing the screening of naïve CD4^+^ T cells entering the LN from the blood for antigen‐specific T cell receptors [205]. Thus, the expression of T_H_2‐tailored molecules and the migratory regulation of CD301b^+^ cDC2s dictate their highly specialized function.

Finally, though cDC1s have been implicated in Treg induction in both the spleen and skin‐draining LNs, several studies have shown that cDC2s, and recently CD301b^+^ cDC2s, prime Tregs. Dermal cDC2s, but not cDC1s, express the enzyme retinaldehyde dehydrogenase (RALDH) to produce all‐trans retinoic acid (RA) [44], a critical positive regulator of Tregs [211]. Another study showed that CD301b^+^ PD‐L2^+^ IRF4‐dependent cDC2s can acquire topically applied antigen through hair follicles, migrate to skin‐draining LNs, and prime Tregs, though the characteristics that facilitate Treg skewing by these DCs were not defined. A recent study demonstrated that in neonatal skin, CD301b^+^ cDC2s preferentially take up and present skin commensal bacterial antigens to T cells, inducing early life tolerance to commensals such as *Staphylococcus epidermidis* [212]. Neonatal CD301b^+^ cDC2s demonstrated elevated RALDH2 production of RA and Treg induction, which is preserved but reduced in adult mice [212]. Therefore, in the absence of type 2 allergic inflammation, these studies demonstrate the capacity for tolerogenic functions of CD301b^+^ cDC2s, indicating a functional flexibility of both cDC1s and cDC2s for priming both pro‐ and anti‐inflammatory T cell responses based on contextual information from the skin.

## 6 DC Migration in the Lung

### 6.1 DC Localization and Migration Kinetics in the Lung

The lung is a specialized organ for gas exchange, and as such has a large surface area that interfaces directly with the external environment. Because of this, the lung environment is subject to many encounters with potential pathogens and inflammatory stimuli, requiring extensive surveillance by DCs [213]. Intravital imaging of DCs in the lung has elucidated their localization, revealing that DCs are primarily localized around airways and in the alveoli, and that the alveolar DCs are relatively stationary, whereas the airway‐associated DCs are more mobile [214]. The lack of movement of alveolar DCs is likely due to their surveillance of the alveolar space, as these DCs produce more dendrites that cross the epithelium than the airway DCs. Upon fluorescent bead administration to mice by inhalation, CD103^+^ migratory cDC1s and CD11b^+^ migratory cDC2s both took up and trafficked beads to the LN. cDC1s and cDC2s are equally represented in the bead^+^ fraction of DCs, despite a lower frequency of migratory cDC1s observed [214]. This is in line with a preference for uptake of particulate antigens such as beads by lung migratory cDC1s, whereas lung migratory cDC2s preferentially uptake soluble protein antigens such as OVA protein [215].

The ability of cDCs to efficiently uptake inhaled fluorescent antigens enables tracking of DC migration to the LN. Similar to the skin, DC migration to the LN has been measured as early as about 6 h post intratracheal administration of antigen, peaking at about 24 h [216]. Additionally, DCs are dependent on CCR7/CCL21 signaling for steady‐state migration from the lung to the mediastinal LN, and partially dependent on CCR8 for LN accumulation [217], further mirroring dermal DC migration. Interestingly, this study was performed in the absence of type 2 inflammation, pointing to a homeostatic usage of CCR8 for LN migration that differs from the skin [203]. Studying the migration of DCs in the lung has the advantage over the skin that both cDC1s and cDC2s take up and migrate with fluorescent antigen at relatively similar ratios, whereas in the skin, cDC2 uptake and migration dominates [109]. Despite this, the kinetics of cDC1 and cDC2 migration have not been extensively compared in the lung, leaving a gap in our knowledge of the rate of cDC subset chemotaxis at homeostasis.

### 6.2 Pulmonary DC Migration to Activate CD8 ^+^ and CD4 ^+^ Effector T Cell Responses

The division of labor in stimulating T cell responses between migratory cDC subsets is largely conserved between the lung and the skin. CD103^+^ migratory cDC1s from the lung are critical in the cross‐presentation of viral antigens to naïve CD8^+^ T cells and clearance of infection [218, 219, 220, 221, 222], though CD11b^+^ migratory cDC2s have been shown to contribute to antigen cross‐presentation [223]. A recent study described an unconventional migration of cDC1s from the lung to the spleen to prime CD8^+^ T cells to influenza virus, resulting in the likely advantageous production of long‐lived memory cells that can surveil the circulation for viral antigens and infected cells [224]. Migratory lung cDC1s are particularly efficient at uptake of apoptotic cells and exclusively express TLR3 [225], making them especially tuned to viral surveillance of the pulmonary environment.

Similarly to the skin, lung cDC2s are implicated in mediating CD4^+^ T cell priming in the context of allergic inflammation. Allergic inflammation has been extensively studied in the lung, given the prevalence of respiratory allergies in humans, and DCs are a critical mediator of this disease. Pulmonary inflammation can result in chronic diseases such as asthma, which presents with T_H_1, T_H_2, or T_H_17‐mediated inflammation on the basis of the cytokines and involved immune cells [226]. cDC2s have been implicated in both T_H_2 and T_H_17 priming in the context of asthma, and thus are critical initiators of allergy [226]. Allergens such as HDM and the fungus *Candida albicans* induce tissue damage and subsequent release of DAMPs and alarmins; critically, these signals activate DC activation and migration to the LN to prime T cells [226]. The alarmin IL‐33 in particular is a critical activator of migratory cDC2s to prime T_H_2 responses [227]. Alveolar epithelial cells (AECs) also produce colony‐stimulating factor 1 (CSF1) in response to allergic inflammation, which activates the migration of cDC2s and is required for allergic IgE production [228, 229]. Upon activation and migration, IRF4‐dependent cDC2s induce the priming and maintenance of inflammatory T_H_2 cells [230, 231, 232, 233].

In addition to regulating T_H_2 priming during lung allergic inflammation, IRF4‐dependent migratory cDC2s have also been shown to be key regulators of T_H_17 development in a lung fungal infection model [234]. It seems contradictory that cDC2s could prime both T_H_2 and T_H_17 responses to lung allergic inflammation, given that these responses are generally mutually exclusive [235]. This may be explained by several factors. CD103^+^ cDC1s in the lung have been shown to suppress T_H_17 differentiation by the production of IL‐2, an anti‐T_H_17 cytokine [236]. Additionally, the heterogeneity of cDC2s allows for two cDC2 subsets to specialize in initiating two separate responses in the lung; pulmonary CD301b^+^ cDC2s were recently demonstrated to migrate and prime T_H_2s in response to fungi, whereas a separate Ly6C^+^ cDC2 population primes T_H_17s [237, 238]. T_H_2‐priming CD301b^+^ cDC2s in the skin produce high amounts of IL‐2 [206], further demonstrating their preference for T_H_2 over T_H_17 skewing across tissues.

### 6.3 Lung cDC2s Initiate T_FH_ ‐Dependent Antibody Responses

Finally, several studies have demonstrated the identity of T_FH_‐inducing cDCs in the lung. By utilizing a DC‐specific *Dock8* knockout, our group demonstrated that the selective loss of lung CD11b^+^ cDC2 migration after intranasal immunization with OVA and LPS leads to a reduction in T_FH_ production and germinal center B cells [109]. This response is completely independent of cDC1s as assessed in a *Batf3* ^−/−^ mouse model. Subsequently, in an HDM model of allergic inflammation, cDC2s display a greater ability to induce T_FH_ cells from an ex vivo co‐culture with naïve T cells [239]. Although these studies point to a critical role for cDC2s in lung humoral responses, further subsetting based on cDC2A/B ontogeny has not been performed in this site. In the spleen, Notch2‐dependent cDC2As have been implicated in T_FH_ priming [167], but it remains to be determined whether these results are generalizable. Why cDC2s are possibly more potent at priming T_FH_ cells in lung‐draining LNs might have to do with their increased expression of CXCR5 and therefore ability to localize to the T‐B border, where T_FH_ cells receive early differentiation signals [109].

## 7 DC Migration in the Intestine

### 7.1 The DC Network in the Intestine

The intestinal tract is a complex interface between the body and the environment that requires tight regulation of transport to allow food and nutrients to pass into the body while limiting microbial transit. The intestine is colonized extensively by commensal bacteria, which are maintained in a symbiotic relationship with the host. However, because of the inherently inflammatory nature of bacterial components due to DC expression of PRRs, the host immune system must develop tolerance against commensals or exclude them from the body entirely. Additionally, many pathogens use the intestine as a site of entry, mandating the recognition of pathogenic microbes from beneficial commensals. As a result of these complicated factors, migratory DCs within the intestine must walk a fine line between the initiation of pro‐ and anti‐inflammatory responses to antigens.

The intestinal epithelium separates the food and commensal‐exposed lumen of the gut from the lamina propria (LP) where many immune cells are localized. Embedded along the small intestine are unique SLOs called Peyer's Patches (PPs), which are organized slightly differently from LNs. They contain T cell zones and B cell follicles underneath a subepithelial dome (SED) containing DCs and other cells in close contact with the epithelium [240]. The LP of both the small intestine and colon is drained by a chain of mesenteric lymph nodes (MLNs). It has been recently described that individual MLNs drain distinct regions of the intestinal tract, and that DCs migrate according to this regional drainage [241, 242].

APCs in the intestine are a heterogeneous mixture comprised of cDCs, moDCs, and macrophages [18]. The DC population in the LP is made up of CD103^+^ CD11b^−^ XCR1^+^ cDC1s, CD103^+^ CD11b^+^ Sirpα^+^ cDC2s, and CD103^−^ CD11b^+^ Sirpα^+^ DCs [9, 243]. In the LP, the CD103^−^ CD11b^+^ DC population is CX_3_CR1^+^, whereas in the MLN, a population with the same surface markers is CX_3_CR1^−^. Both of the CD103^+^ DC populations are true cDCs, as they are dependent on Flt3L for their differentiation, whereas the CD103^−^ DC population is a heterogeneous mix of cDC2s and moDCs, and is dependent on both Flt3L and M‐CSF [9, 244, 245]. Additionally, the CD103^+^ CD11b^+^ cDC2 population is dependent on Notch2 for its development, aligning these cells with cDC2As in other tissues [12]. These populations are consistent between the LPs of the small intestine and colon, though the small intestine has more CD103^+^ CD11b^+^ cDC2s compared to the colon [241]. In the PP, cDC1s are CD8α^+/−^, CD103^+^, CD11b^−^, and XCR1^+^, whereas cDC2s are CD103^−^, CD11b^+^, and Sirpα^+^ [243, 246]. This heterogeneous mix of markers and anatomical locations makes the identification of specific DC subsets difficult, especially when retrospectively examining earlier studies. This is somewhat mitigated by the consistent usage of the integrins CD103 and CD11b across many studies, which minimally stratifies the migratory cDC subsets in the LP.

### 7.2 Homeostatic Surveillance and Antigen Acquisition by Intestinal DCs

DCs in the intestine migrate at steady state from the LP to the MLN, and this process is dependent on CCR7 as in other tissues [9, 247, 248]. Unlike other sites, gut cDC2s co‐express CD103 and CD11b; therefore, many decades of work identifying cDCs with CD103 need to be interpreted carefully regarding the cDC subset implicated. Notably, both CD103^+^ cDC1 and cDC2 subsets are the primary migratory DCs from the LP to the MLN, as the CD11b^+^ CX_3_CR1^+^ population of moDCs is not thought to migrate out of the LP [9, 249]. However, one study indicated that under conditions of commensal bacterial dysbiosis, this DC population can migrate to the MLN via CCR7 and carry bacteria for presentation to T cells, inducing an inappropriate commensal‐specific immune response [250]. The migration of CD11b^+^ CX_3_CR1^+^ DCs is restrained by microbial sensing, as deletion of MyD88 also leads to more DC migration [250]. The migration of CD103^+^ cDCs is also dependent on MyD88 at steady state, though the depletion of the microbiome with antibiotics or germ‐free housing did not impact homeostatic cDC migration, indicating that bacterial PAMPs may not be the primary maturation factor to induce gut cDC migration [251]. In the PP, it is not well understood whether cDCs migrate at steady state, but upon oral immunization they relocate from the SED to the interfollicular zone, co‐localizing with T cells [252, 253, 254, 255]. It has been suggested that LP DCs can migrate to the PPs after administration of an oral adjuvant [256], though this has not been directly demonstrated because of the technical difficulty of delivering antigen specifically to LP DCs vs. PP DCs.

The route and mechanism by which intestinal cDCs acquire luminal antigens prior to migration and T cell priming is a topic of debate in the field. The generally agreed‐upon mechanism for PPs is uptake of luminal antigens via a specialized epithelial cell called a microfold cell (M cell). M cells transcytose antigens, including whole bacteria, across the epithelium into the SED, where transported antigens can be taken up by SED DCs [257]. M cells also contain transcellular pores that DCs have been shown to reach through to directly take up antigens from the lumen [258]. The antigen delivery mechanism for LP DCs is less clear, given that the intestinal epithelium outside of PPs generally lacks M cells. CX_3_CR1^+^ DCs express tight junction proteins, and have been imaged extending long dendrites between epithelial cells to sample bacteria and other luminal antigens [259, 260, 261]. Dendrite formation is dependent on PRR sensing of ligands on epithelial cells [261]. Upon uptake of antigen, CX_3_CR1^+^ DCs can transfer antigen to CD103^+^ cDCs, which are more efficient at migration and T cell stimulation in the MLN to induce tolerance [262]. Gap junction proteins are critical for the transfer of antigens between cells. However, this pathway is disputed by groups that have used intravital imaging to visualize fluorescent uptake in live anesthetized animals. Through this imaging method, several studies have demonstrated antigen uptake and transcytosis to the LP by goblet cells, a specialized epithelial cell that is the major mucus‐producing cell in the gut [263, 264]. These goblet cell‐associated passages (GAPs) hand off antigen directly to CD103^+^ cDCs in the LP but not CX_3_CR1^+^ DCs [263]. GAPs are primarily active in the small intestine, as microbial sensing in the colon, where microbiome abundance is at its highest, shuts off antigen uptake [265, 266]. Although the relative usage in vivo of transepithelial dendrites vs. GAPs for cDC antigen acquisition is still an open question, both mechanisms have been demonstrated to mediate tolerance to food antigens and commensal bacteria in a cDC‐dependent manner [262, 266, 267], demonstrating the importance of regulating migratory LP cDC antigen uptake.

### 7.3 Intestinal Migratory DCs Are Critical Mediators of Oral Tolerance

Although migratory cDCs have been extensively shown to regulate pro‐inflammatory T cell responses in the gut by priming T_H_ and T_FH_ cells as in other tissues, their role in establishing oral tolerance is perhaps their most unique function in the intestine compared to other sites. Tolerance against both food antigens and commensal bacteria is one of the most important immunological functions of intestinal DCs, and is primarily dependent on CD103^+^ cDC migration from the LP to the MLN [248, 268, 269, 270]. Extensive literature has characterized the ability of LP migratory cDCs to induce Tregs and oral tolerance, and has demonstrated that cDCs can produce RA with the RALDH2 enzyme and activate latent TGFβ with αvβ8, both of which are critical signals for Treg induction (reviewed by Liu et al. [18]). Interestingly, more abundant tolerogenic Tregs are induced by migratory cDCs in the MLNs draining more proximal regions of the small intestine, such as the duodenum [242], which correlates with earlier antigen encounter after ingestion. The production of RA and activation of TGFβ is not exclusive to cDC1s or cDC2s, creating uncertainty as to which cDC subset is critical for the induction of tolerance. To address this, a recent study used the LIPSTIC model of proximity labeling to identify the DC interactions that give rise to Tregs after oral antigen encounter. The study found that migratory but not resident cDC1s and cDC2s present antigen to naïve T cells after antigen administration, but that the migratory cDC1s are more capable of inducing antigen‐specific Tregs [271]; in contrast, migratory cDC2s induced more T cell anergy. However, co‐infection with helminths abrogated the induction of oral tolerance by inducing an influx of cDC2s to the MLN, out‐competing tolerogenic cDC1s for T cell interactions to produce helminth‐specific T_H_2s [271]. Thus, the authors propose that cDC1s are the primary cDC subset that induces Tregs against oral antigens, and that the ratio of cDC1s to cDC2s determines the balance of tolerogenic vs. inflammatory T cell responses produced in a competing gut environment.

Despite the abundance of data pointing to a critical role for migratory intestinal cDCs in promoting Treg induction and tolerance, recent evidence suggests that a novel subset of RORγt^+^ APCs is a critical mediator of tolerance to both commensal bacteria [272, 273, 274, 275], as well as oral food antigens [275, 276, 277, 278, 279]. These APCs express αvβ8 to activate latent TGFβ, require MHC‐II to present antigen to CD4^+^ T cells, and are particularly enriched during early life. RORγt APCs require CCR7 expression for Treg induction [274], indicating a strict migratory requirement to carry out their Treg priming function. Gavage of fluorescently tagged OVA protein demonstrated that RORγt APCs are able to take up antigen and migrate to the MLN, appear as early as 8 h post‐gavage, and arrive at the MLN before migratory cDC subsets [277]. The developmental lineage of RORγt APCs is currently debated, with some groups suggesting a myeloid lineage, whereas others suggest that they arise from lymphoid precursors [280]. Regardless of their relation to cDCs, they likely cooperate with cDCs to induce optimal tolerance in the intestine [276]. These findings add an additional layer of complexity to the already confounding gut environment and underly the necessity for a full understanding of the regulators of tolerance and how they become dysregulated during allergy or dysbiosis.

## 8 Conclusion

Dendritic cell migration is an essential process that allows the spatiotemporal transmission of contextual information from peripheral tissues to lymphoid organs. DCs act as sentinels in tissue and patrol various sites in the body for anything that the many PRRs on DCs might signal as harmful; if none are encountered, foreign (e.g., food) or self‐antigens that are sampled by DCs are presented to T cells to enforce tolerance after steady‐state migration to SLOs. However, if DCs sense inflammation and become activated, they engage in rapid, directional migration to SLOs to prime a T cell response that matches the pathogen or stimuli that were encountered. In this way, the migratory process of DCs functions as a funnel for a wide variety of signals to bring peripherally derived antigen to a cognate naïve T cell in lymphoid tissue. Even after reaching the splenic WP or the LN parenchyma, DCs utilize various chemotactic gradients to fine‐tune their position for optimal presentation of antigen to the T cell niche that suits the proper immune response. As such, the process is highly regulated to ensure that antigen delivery and T cell stimulation are efficient while also as accurate as possible.

The intracellular events that induce DC migration are complex, involving simultaneous activation of several signaling cascades downstream of chemokine receptors. These cascades result in cytoskeletal rearrangement to drive ameboid migration through tangled 3D environments, with dynamic polymerization and depolymerization of actin and tubulin filaments, as well as nuclear pathfinding to prevent cellular entanglement. We now understand much about these processes, though there is still much left to understand. As recent studies have revealed how diverse and heterogeneous the DC lineage is, it will be important to understand whether what we already know about signaling and cytoskeletal organization during migration is translatable across different DC subsets.

The outcomes of DC migration are varied, resulting in either the establishment of tolerogenic T cell responses or several different types of inflammatory T cell responses, each characterized by a distinct cytokine and effector cell profile. As we study the outcomes of DC migration in various tissues and with different types of pathogens and adjuvants, it becomes increasingly clear that cDCs accomplish adaptive immune initiation through a division of labor. For example, across tissues, it is consistent that cDC1s are particularly well‐suited to prime CD8^+^ T cells and T_H_1 cells, whereas CD301b^+^ cDC2s (or analogous tissue‐specific cDC2Bs) are effective T_H_2 priming DCs. Notch2‐dependent cDC2As are efficient at T_H_17 priming, and perhaps the best at inducing T_FH_ and humoral responses, although this aspect still requires more exploration. Many different migratory cDC subsets, however, can induce Tregs in a wide variety of contexts, including the newly discovered RORγt APCs. It is additionally unclear whether the preference of specific DC subsets for each type of response changes based on the type of adjuvant or pathogen used in a particular model, as there are few studies that attempt to compare the ability of a specific cDC subset to induce different T cell responses given different stimuli (type 1 vs. type 2 vs. type 17). Indeed, there is a large degree of redundancy for many DC functions, and even in the most clear‐cut cases of preference, such as cDC1s for CD8^+^ T cells, cDC2s can cross‐present many types of antigens if cDC1s are absent or inhibited. Future work should attempt to define the functional heterogeneity of migratory cDCs and to what degree specialization in a given response is strict or flexible. Additionally, as a key checkpoint for long‐lived adaptive immune responses, DC migration presents an attractive target for both the initiation and inhibition of inflammation, depending on the therapeutic context.

## Conflicts of Interest

The authors declare no conflicts of interest.

## Data Availability

No data was generated in this study.
